# Measurement of gender as a social determinant of health in epidemiology—A scoping review

**DOI:** 10.1371/journal.pone.0259223

**Published:** 2021-11-03

**Authors:** Céline Miani, Lisa Wandschneider, Jana Niemann, Stephanie Batram-Zantvoort, Oliver Razum

**Affiliations:** 1 Department of Epidemiology and International Public Health, School of Public Health, Bielefeld University, Bielefeld, Germany; 2 Institute of Medical Sociology, Martin-Luther University Halle-Wittenberg, Halle, Germany; University of Perugia: Universita degli Studi di Perugia, ITALY

## Abstract

**Background:**

The relevance of gender as a social determinant of health and its role in the production of health inequalities is now broadly acknowledged. However, the plethora of existing approaches to capture gender, which often stem from disciplines outside of epidemiology, makes it difficult to assess their practicality and relevance for a given research purpose. We conducted a scoping review to 1) map the evidence of how gender can be operationalised in quantitative epidemiology and 2) design a tool to critically evaluate the measures identified.

**Methods:**

We identified peer-reviewed articles in electronic databases (PubMed, Embase and PsycINFO). Eligible sources described the quantitative operationalisation of the social dimension of gender. With the help of a newly developed checklist, we assessed their relevance from an analytical perspective (e.g. intersectionality) and their potential for implementation in epidemiology.

**Results:**

Gender measures principally assessed gender roles and norms, gender-based discrimination and violence, and structural gender (in)equality. Of the 344 measures included in this review, the majority lacked theoretical foundation, and tended to reinforce the binary understanding of gender through stereotypes of femininity and masculinity. Only few measures allowed for an intersectional approach and a multilevel understanding of gender mechanisms. From a practical point of view, gender measures demonstrated potential for use in varied populations and contexts.

**Conclusions:**

A range of gender measures are readily available for epidemiological research, addressing different levels and dimensions of gender as a social construct. With our theory-informed, practice-driven scoping review, we highlighted strengths and limitations of such measures and provided analytical tools for researchers interested in conducting intersectional, gender-sensitive analyses.

## Background

### Gender as a social determinant of health

Gender is conceptualised as one of the core social determinants of population health and health inequalities within the social determinants of health (SDH) framework of the World Health Organization (WHO) [1]. In this context, gender should be understood as an individual’s socially ascribed attributes, roles, responsibilities, and expectations in a given society based on their gender expression and how others perceive it (in contrast to sex being about the biological, physiological, genetic and hormonal bodily characteristics of a person) [2]. Rooted in feminist sociology, this approach conceptualises gender not merely as an individual’s trait or identity but as a social system allocating differential resources and positions to men, women and gender diverse individuals. Social processes, such as discrimination, social sanctioning or confirmation, produce and maintain social systems of norms and power hierarchies [3], both within and between gendered groups as the analytical lenses of hegemonic masculinities [4] and intersectional feminism [5] suggest. These social processes operate simultaneously at different levels and dimensions -ranging from the policy and organisational level to the private sphere and from formal, clearly observable (e.g., the commitment to the Sustainable Development Goals (SDGs)) occurrences to rather informal and unspoken rules and norms (e.g. moral prejudices).

Together with other social determinants of health, gender shapes health outcomes through the differential exposure to intermediary determinants of health, i.e. material (housing, neighbourhood quality, consumption potential), psychosocial (coping styles, stressors, relationships) or behavioural and biological factors. Gender, and all the concepts that it relates to (e.g. masculinity, femininity but also patriarchy, sexism, and heteronormativity), can have protective or detrimental effects on health through different pathways, including differentiated risk exposure, gendered behaviours, use of and access to healthcare services, and gender bias in health systems [3].

In spite of the growing awareness of how gender influences health, gender is still often conflated with sex in health research [6–8]. This is especially the case for quantitative analyses in epidemiology or health reporting/monitoring where gender analysis sets out to compare men’s and women’s health statuses and behaviours, while relying mostly on sex-stratified or sex-specific approaches and an essentialist, binary understanding of gender [9]. The sex-specific approaches are a necessary step towards the understanding of health inequalities, but they are not sufficient [10], the use of two categories (male/female or men/women) for sex and/or gender identification having shown its limits [10,11]. They may even incorrectly convey the idea that sex and gender can be used interchangeably—in spite of representing distinct constructs—which ultimately compromises the validity of research [12]. Although a social phenomenon which is difficult to grasp through quantifiable aspects, gender, in all of its diversity and dimensions, has somehow to be operationalised, i.e. measured and quantified in the context of epidemiology, to expose and deconstruct the mechanisms that contribute to and maintain gender-related health hierarchies.

### (Theoretical perspectives on) Conceptualising gender in epidemiology

Epidemiology has always studied gender-related health inequalities (e.g. SDGs monitoring [13] and the use of the Demographic Health Surveys (DHS)[14,15]) and in recent years there have been renewed efforts to incorporate more theory into quantitative analyses. More specifically, in relation to questions of equity and social power, intersectionality theory has gained popularity in research on gender and health over the last few years [16–18], as it emphasises the multilevel complexity of power relations and the connectedness of different social positions. Intersectionality theory originated from critical race and Black feminist theory and practice, in particular the Combahee River Collective Statement [19], as Kimberlé Crenshaw used it to challenge the exclusion of black women and men from white feminist and antiracist discourse [5]. It argues that the different social positions of an individual such as race, gender, sexual orientation, education, socioeconomic status, and (dis)ability intersect at the individual level to create inequalities, but are ultimately the product of interlocking systems of marginalisation, oppression, and privilege such as racism, classism and sexism.

This perspective aligns well with epidemiological theories on population health distribution, like the ecosocial model by Nancy Krieger [20]. This embodiment approach helps to make explicit how social, political and environmental contexts physically incorporate into peoples’ bodies over time (intergenerational and lifetime-course) and space (on global, national, regional or household level) and thereby affect individuals’ biological and physiological processes. As such, Krieger introduces a multilevel framework that sheds light on potential associations between exposures and health outcomes [21].

While Krieger’s perspective clearly exposes the pathways to embodiment by disclosing the ‘cumulative interplay’ between exposure, resistance, and susceptibility, the intersectional approach focusses on the processes of power, privilege and oppression that determine an individual’s social position. Translating intersectionality theory into epidemiology means understanding social positions as determinants of health, and their intersection as the inter-dependent, multi-directional effects they have on each other and on health outcomes. Despite the multiple commonalities between the ecosocial and intersectional approaches [22], we decided for the framing of this review to adopt principally an intersectional perspective as our focus lies explicitly on gender as a health determinant (and not, as an embodiment approach would suggest, on the distribution of diseases). Moreover, intersectionality theory has been at the core of groundbreaking methodological developments in epidemiology in recent years, and seems more fitting to our focus on quantitative analysis (e.g. [17,18]).

Applying an SDH and intersectional lens on gender emphasises that gender-related health inequities are linked to social, political and economic factors rather than being limited to individual and behavioural ones. It thereby contributes to take into account upstream causes that shape each individual’s opportunities, shifting the responsibility from the individual to societal power structures. Such endeavor has the potential to move research from gender-sensitive terrain to gender-transformative action, which is in line with intersectional approaches seeking social change. Beyond the acknowledgement of gender differences in gender sensitive approaches, scholars have indeed been calling for years for gender transformative research that aims to challenge “existing gender norms and power structures to reduce gender inequities while also accounting for how other embodied and ascribed identities influence these norms”,[23] and “seek to move beyond individual-level change and instead centre on restructuring the power relationships that create and maintain gender inequalities” [7]. Especially researchers in the field of health promotion have investigated the potential of gender-transformative interventions [24–26]. In terms of measurement and in the field of social epidemiology, gender-transformative research could mean a better understanding and integration of social inequalities in instrument design, allowing to capture discriminatory practices and power dynamics [27], a focus on the views and lived experiences of marginalised groups (e.g. women [28], or gender diverse people), or surveying groups on topics they’ve been excluded from so far (e.g. research on masculinities and reproductive health, including men and abortion stigma [29]). Gender-transformative epidemiological research would also be “gender expansive”, promoting gender diversity and inclusivity and challenging the traditional normative dichotomy opposing men and women, the masculine and feminine [30].

### Rationale and objectives

Hitherto no overview of gender measures in epidemiology from an SDH, intersectional perspective exists. In the fields of psychology and sociology, some recent reviews have focused on specific gender dimensions and their quantitative operationalisation, e.g. masculinity [31], gender inequality at a macro level [32–34], and sexual and gender minorities (SGM) discrimination experiences [35]. There are also public health oriented overviews of the different types of gender measures [36], or of a specific type of measures (e.g. on secondary data analysis [37]), and reviews of implementation and policy-oriented frameworks and tools [38]. Here we proposed to combine (i) a critical, theory-informed analysis of the types of gender-related measures one can use in epidemiology with (ii) a thorough description of the measures, and (iii) a practical tool to guide the choice of measure in future research. Doing so, we aimed to answer the following questions:

- What are the quantitative measures already available to epidemiologists seeking to assess gender as a social determinant of health?
- How do those measures operationalise gender and which dimensions and levels of gender do they investigate?
- Which measures are best suited for different research questions, taking into consideration how they operationalise gender in terms of analytical potential and practical implementation?

## Methods

We conducted a scoping review to assess the ways in which gender as a social determinant of health can be measured in epidemiology. A scoping review aims to map the range of evidence of a specific research area, identify potential gaps and synthesize knowledge [39,40]. Unlike systematic reviews, it allows for broader scope and iterative approaches, useful to identify the nature and extent of ongoing research in all formats (e.g. grey literature) [39]. Last, scoping reviews have been used to examine not only *which* but also *how* research has been conducted, which fits our intention to assess the actual measurement of gender [39]. We followed the methodological steps of the PRISMA-ScR protocol [41], therefore ensuring transparency and replicability (S1 File), but did not publish a review protocol.

### Multilevel approach to gender analysis in epidemiology

**Fig 1:**
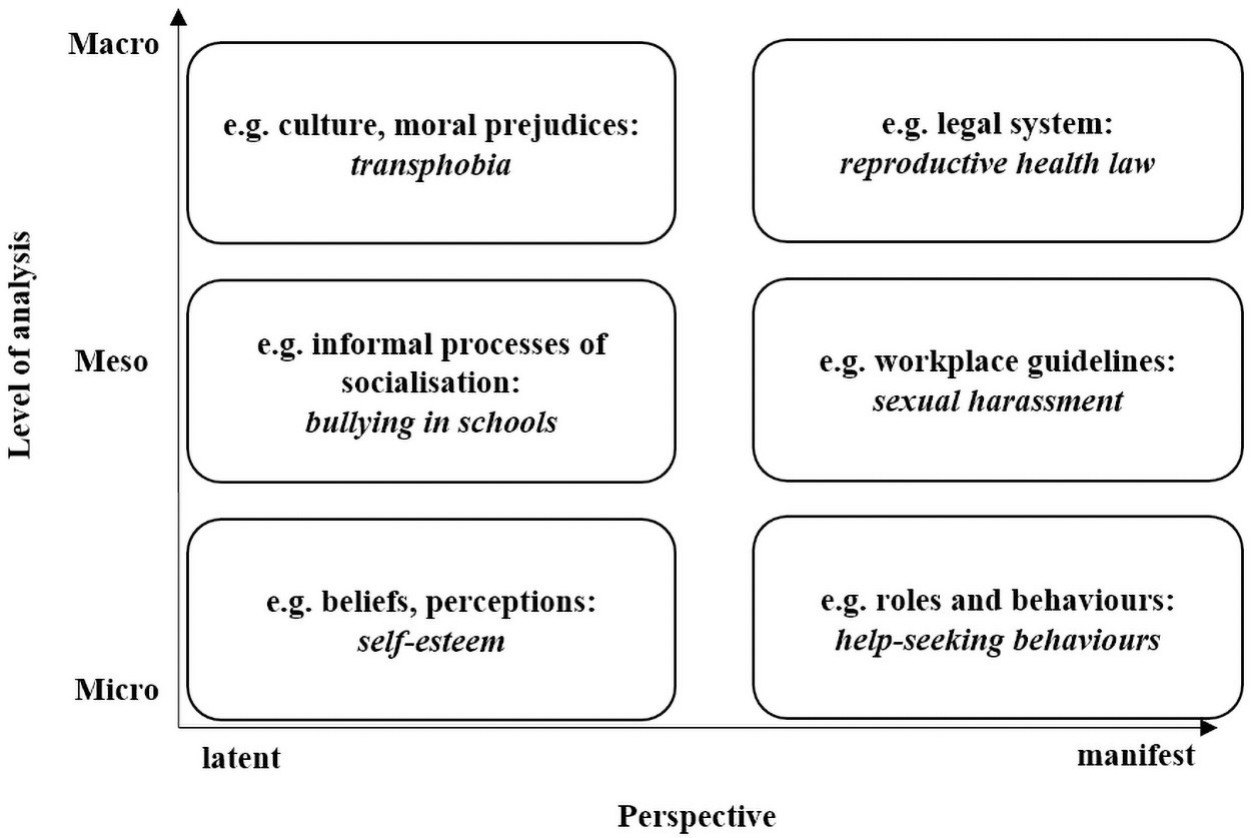
Multi-level approach to understanding the latent and manifest gender-related determinants of health. Source: Authors’ own elaboration, adapted from Ritzer [43].

To guide our methodological approach and the analysis, we applied a multilevel approach to gender analysis in epidemiology (Fig 1). We grouped factors contributing to shaping gendered health outcomes into three levels that are commonly used in health system analysis (e.g. Jhpiego framework[42]):

- the structural (macro) determinants of gender inequalities are located at the state-level and include policies, institutions and legal frameworks;
- the organisational (meso) determinants address the community and organisation level encompassing e.g. health care services, neighbourhoods and community facilities;
- the individual level (micro) factors relate to the experiences of individuals themselves or in relation with others, such as their partner, friends, family or children.

These levels of analysis differentiate further between latent and manifest dimensions. The manifest mechanisms that influence health have a formal, measurable or objectified character. At the macro level, legislations promoting gender equality could serve as an example of manifest gender mechanisms. Manifest meso-level examples pertain to guidelines or policies in healthcare facilities (e.g. on the inclusion of SGM patients), and micro level examples include for instance insults as a measurable expression of gender stereotypes and discrimination. The latent gender mechanisms are underlying and informal, yet highly effective, e.g., cultural norms and stereotypes of a particular society at the macro level; collectively shared patterns of interpretation in a specific facility or community (meso level), or power dynamics (e.g. discrimination) that shape perceptions of everyday lived experiences at the micro level.

### Search strategy

We aimed to identify measures operationalising gender in quantitative analyses in epidemiology, rather than individual studies. We considered “measure” any instrument, scale, index, questionnaire, or scoring that combines a set of variables to build a score or categories that quantify in a way or another gender as a social determinant of health. To retrieve those measures, we searched the peer-reviewed literature in the databases PubMed, Embase and PsycINFO. The original search was conducted on 19.11.2019 and an update on 11.02.2021. The research team developed the search strategy with the support of an experienced librarian. The terms for the concept of gender encompassed a variety of gender dimensions, referring to gender roles, norms, inequalities and discrimination. They reflected the current state of research in the field of sociology (whose concepts framed our approach to gender, see Background) and epidemiology (the discipline where the review is located). We also checked against terminology used in advocacy and activism websites and discussed in conversations with gender experts. As gender discrimination is often closely linked to heteronormative views on sexual orientation, terms related to sexual orientation and sexual and gender minorities were also included in the search. To restrict the findings only to examples of measurement strategies in epidemiology, we added terms such as measure, measurement, tool, scale, index or instrument (S2 File).

In addition to the database searches, we screened measures that were referred to in the ones we included through a snowball process, and conducted web-based searches to identify the original or updated versions of a same measure.

### Eligibility criteria and selection

To be included in the review, the measures needed to describe the quantitative operationalisation of gender as a social determinant of health. We did not include measures if:

1. they did not fit into the analytical framework of the scoping review, e.g. because they only referred to sex assigned at birth, or were clinical tools to inform gender identity related healthcare decisions. On this basis, we also excluded the growing literature on categories for self-defining gender in surveys (e.g. [11,44,45]) which seeks to assess the most ethical and practical way to capture diverse self-reported gender identities in quantitative health surveys [46]. The focus of this research is on counting and including different categories of individuals, rather than on capturing the processes that lead to gender-related health differences across those categories.
2. they were not well described (e.g. missing information on items or score calculation). We limited the search to the years 2000 and later to capture the most recent developments in the field. Doing so, we were still able to include measures developed before 2000, as long as they had been used in the past 20 years, or served as reference for the measures already included. We did not apply any restrictions with regard to language, geographical region, or a minimal number of applications of the measure.

To increase consistency among the reviewers who conducted the screening (CM, LW), we independently screened a random sample of 5%. We then discussed potential inconsistencies and, if necessary, modified the eligibility criteria. Afterwards, the two reviewers started to screen the titles and abstracts of the published literature and continued with the full-texts of potentially relevant papers. Disagreements on the inclusion of specific measures were resolved by discussions to achieve consensus, if necessary, with a third reviewer (JN).

### Data charting

Data extraction and charting pertained to the measures, rather than to articles screened. Accordingly, there is only one entry in the extraction table about each measure, drawing from the information contained in the initial article describing the measure. When needed (e.g. when the initial article did not display the full list of items that made up the measure), we used more recent articles that included relevant information on the measure or presented updated versions. The descriptive data charting followed a standardised form, which was jointly developed by the reviewers (CM, LW) (Table 1). CM, LW and JN extracted the data relating to the instruments included, discussed the results and updated the charting form in an iterative process. The form captured data on article characteristics (e.g. author, name of the measure) and details about how gender was operationalised, e.g. what gender aspects were considered, what specific indicators were used and in which dimension/levels gender were conceptualised.

**Table 1:** Data charting form.

Category	Description
Name of the measure	As stated by the authors
Reference	Full reference of the original article describing the measure
Date of creation/first use	When was the measure created or used for the first time?
Region of origin	In which country/for which context has the measure been developed?
Short description	What is measured? What are its aims?
Based on (if applicable)	Is the measure based on another measure? If so, state the author and tool
Gender domain	Indicate which gender domain the instrument principally pertains to (e.g. beliefs and perceptions, access to and control over assets^1^)
Level of analysis (see framework, Fig 1)	Indicate the level that is intended to be operationalised (macro, meso, micro level; manifest/latent dimension)
Items	Examples of items or variables included in the measure
Revisions of the instruments	Are revised versions of the measure available?
Study population and study participants (when applicable)	Indicate the populations who are targeted in the measure and those who are surveyed
Survey instrument vs. methods for secondary data analysis	Classify as a survey instrument (primary data collection) or a method feasible for secondary data analysis
Validation of the measure (when applicable)	Have the psychometric properties of the measure been validated?

Note:

^1^ Gender domains according to the Jhpiego gender analysis framework [42].

### Analysis

The data charting form and the multilevel framework introduced above guided the descriptive synthesis of the measures. In addition to the descriptive synthesis, we sought to appraise critically the measures included in the review through the development of a practical tool, a checklist that allows reflecting on the characteristics of the gender measures (Table 2). The checklist items are organised in two main categories: their relevance from an analytical perspective and their potential for implementation from a practical point of view. The analytical items are a translation of the key gender concepts that we described in the background and which make up for a current, state of the art understanding of gender (e.g.[16])–namely, inclusion, intersectionality, multilevel understanding and transformative potential (see details in Table 2). Among the practice-based items, we took into consideration the length or complexity of the measure, its (internal) validity, and its transferability. The development of those items was guided by reviews of best practice for the design and use of questionnaires in health research [47,48]. The relevance of each item varies, depending on the research question that scholars want to investigate, which is why we neither provide scores nor do we claim to give an extensive and holistic evaluation. The checklist does not constitute a quality assessment tool. It rather intends to capture the multiple demands in recent gender-sensitive research. Through it, researchers can better visualise which instrument would fit their purpose, depending on the items that are most important for a specific research question. For example, researchers may look specifically for instruments which present alternatives to the dichotomous men/women variables, or for instruments that can be used for multi-country comparisons.

**Table 2:** Checklist to guide reflection on gender measures.

**Analytical items**	
Inclusion	Many gender measures are bound to oppose men and women, or uniquely rely on views on masculinity and femininity. Inclusive measures would be those that capture the fact that additional types of gender identity and expression exist, which can be reflected in more nuanced norms and roles. *Does the measure rely on a non-binary understanding of gender*?
Intersectionality	Intersectionality theory argues that the different social positions of an individual such as race, gender, sexual orientation, education, socioeconomic status, and (dis)ability intersect at the individual level to create inequalities. *Does the measure seek to capture the intersection or interaction of several social determinants of health*? *Is gender considered in relation with at least another social determinant of health (e*.*g*. *socio-economic status*, *ethnicity)*?
Transformative potential	Gender-transformative research seeks to challenge prevailing gender norms and hierarchies and contribute to social change and gender equity. *Does the measure avoid reproducing gender stereotypes*, *including heteronormative prejudices*? *e*.*g*. *is the measure “symmetrical”*, *in the sense that participants of all genders are equally asked about their roles as parents*?
Multilevel understanding	Gender-related social processes operate at different levels (macro to micro) and dimensions (latent, manifest). *Does the measure address different levels of gender processes*, *and within the levels*, *different dimensions*? *Or is it focussed on one level*, *and/or one dimension*?
**Practice based items**	
Length and complexity	Practical considerations such as data availability and resources allocated to data collection may lead to asking the following questions: *If using secondary data*: *does the measure include many variables*? *How specific are those variables (e*.*g*.: *full-time vs*. *part-time work or number of hours worked per week*?*) and can they be substituted for equivalent variables/proxies*? *If the measure requires primary data collection at the individual level*: *Is the questionnaire long*? *Is there a shorter version*? *Would the burden on participants be reasonable*?
Validity	Validity signifies that the instrument actually measures what it claims to measure. *Has the measure been validated*? *If yes*, *in which context*? *Among which population sub-groups*?
Transferability	Being able to compare findings to other studies’ findings, or to do multi-settings analyses, require the use of measures that are transferable, or generalizable. *Is the measure available in several countries*?*; or is it adaptable to different contexts*? *Has it been translated in different languages*?

## Results

The initial search in the databases retrieved 5085 papers (duplicates removed). We retained 1550 papers from which we excluded 117 in a full-text screening step as they did not provide a quantitative measure (n = 9), did not investigate gender as a social determinant (n = 83) or because the full-text was not available (n = 15). Full-text screening and snowball searches (see Method section) led to the identification of 344 measures (S3 File). The measures were developed between 1968 and 2021, with more than half published after 2010. Most of them originated in the United States (n = 226) (S4 File).

### What do the measures capture?

**Fig 2:**
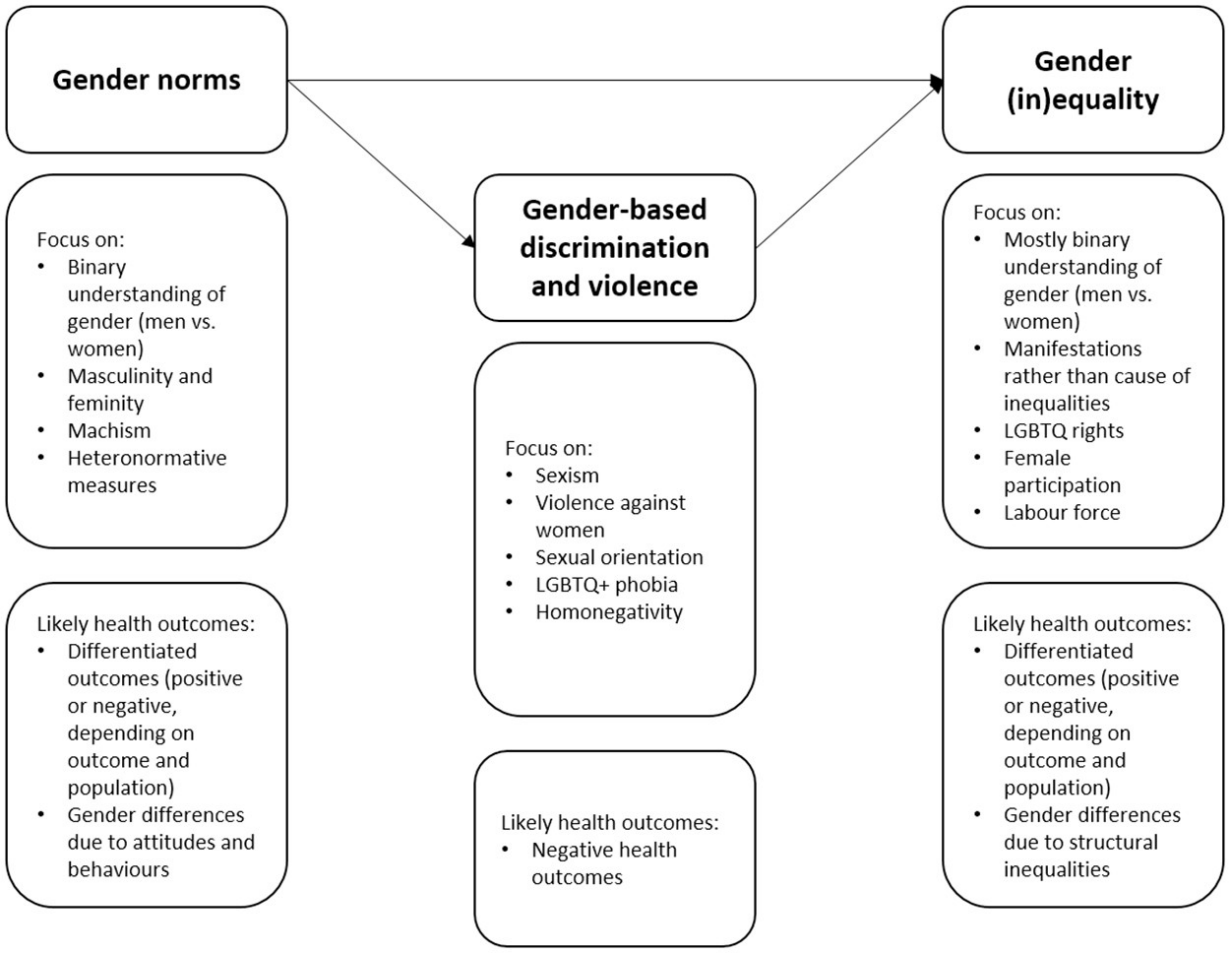
Categories and main characteristics of gender measures. Source: Authors’ own elaboration.

Fig 2 summarises visually the types and characteristics of gender-related measures identified in the quantitative health literature.

The 344 gender measures can be broadly divided in three categories: gender norms (n = 139), gender-based discrimination and violence (n = 145), and gender (in)equality (n = 60). The allocation of measures to those categories runs the risk of oversimplifying the gender measurement landscape, but helps provide an overview of the main orientations of gender research so far. The three categories can be articulated in a sequential schema, with the norms being the main cause of the discrimination and (in)equality, and discrimination reinforcing (in)equality.

The thematic focus was similar in the categories “gender norms” and “gender (in)equality”: both exhibited predominantly a binary understanding of gender (men vs. women) and explored the causes or consequences of masculine and feminine norms in a heteronormative world. Health outcomes tended to be unfavourable for men or women, depending on the type of outcome (e.g. suicide risk, self-rated health) and influenced by ascribed roles in society for women and men. Additionally, few instruments in these categories related to SGM identity and community belonging, assessing their positive or negative dimensions on the lives of SGM population groups (e.g. [49,50]).

The focus was different in the gender-based discrimination and violence category, with an emphasis on groups who were systematically disadvantaged within societies dominated by heteronormative and patriarchal norms. Most of the measures were about sexism and violence against women on the one hand, and LGBTQ+ phobia and discrimination on the other hand. In studies using these measures, most health outcomes showed the negative impact on health of discrimination and violence.

With regard to how the measures were constructed, the categories “gender norms” and “gender-based discrimination and violence” had in common that they tended to focus on the experiences, perceptions and opinions of individuals. Those were collected at the micro-level through the deployment of surveys. They were usually made up of a series of items (e.g. “Someone assumed that I want children because of my gender”[51]), sometimes divided in thematic categories (factors or dimensions, e.g. sexist language, sexual objectification [51]), and rated with Likert scale.

The third category (gender (in)equality) was different in nature: it was not about what causes inequality but how inequality manifests in society, in different spheres of life (e.g. politics, economy, health). The analysis was almost exclusively at the macro-level, using aggregated data which reflected structural (in)equality. Those measures of gender (in)equality were often used to monitor progress towards equality and improved outcomes. In most cases, they stemmed from the fact that inequality exists in disfavor of women compared to men. Exceptions to this were measures which assessed the existence of protective or discriminatory legislation toward SGM (e.g. ILGA Rainbow Map on national legislation towards sexual minorities [52] and the Sexual Orientation and Gender Identity (SOGI) Human Rights Index [53]). Gender (in)equality measures were often created by international organisations (e.g. Social and Institutional Gender Index by the OECD [54] and the Gender Inequality Index by the UN Development Programme [55]), using data routinely collected in a range of countries. A few measures focused on promotion of gender equality at the meso-level, e.g. in the workplace or in schools [56,57].

**Fig 3:**
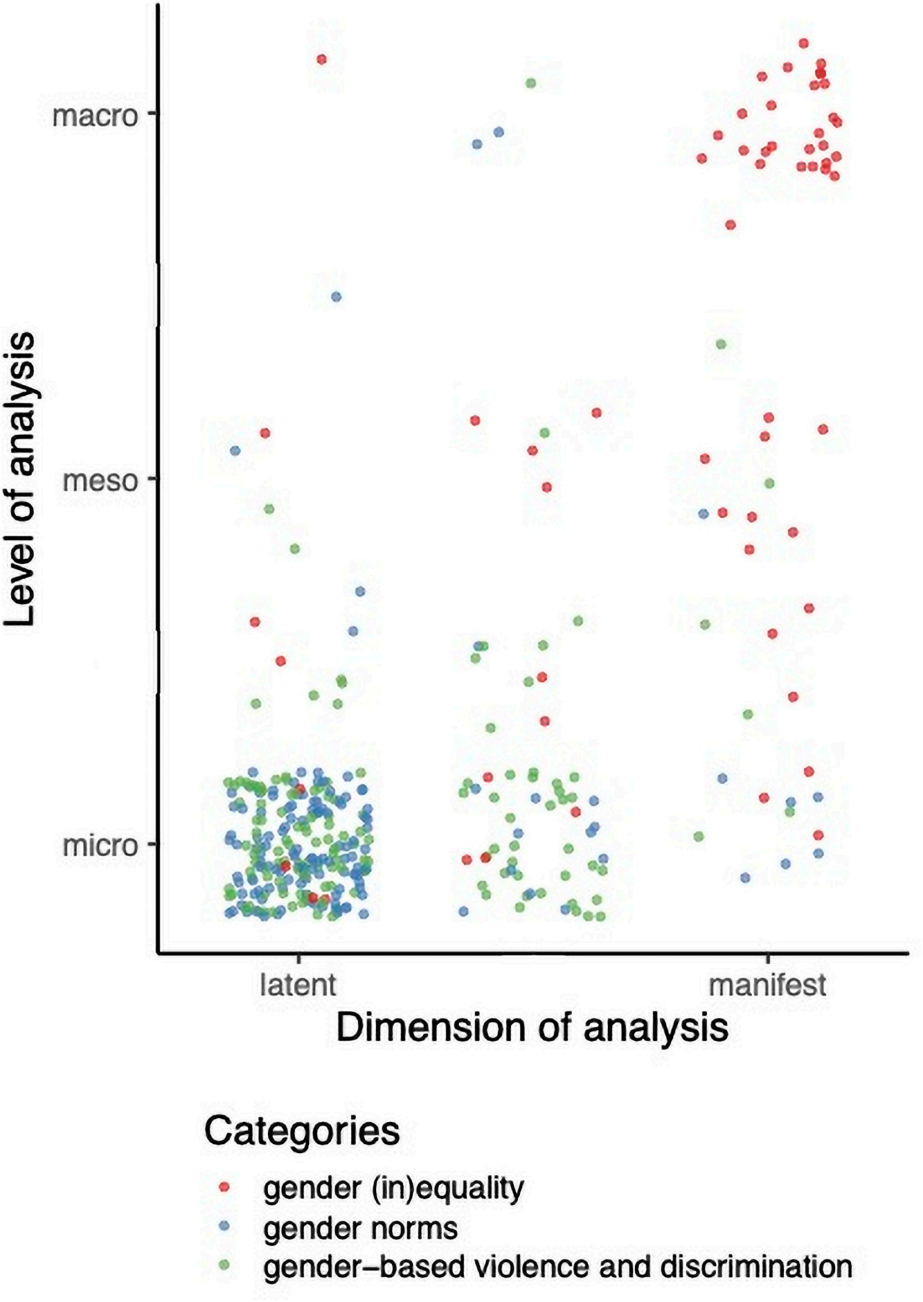
Multilevel mapping of gender measures used in epidemiology. Source: Authors’ own elaboration.

Fig 3 highlights how the different measures mapped against our multi-level framework. The gender norms measures tended to focus on the latent micro-level; the discrimination and violence measures also addressed the micro-level, mostly in a latent dimension; and the gender inequality measures investigated predominantly the macro-level, in a manifest dimension. This is partly linked to the data collection approach and the afore-mentioned dichotomy between survey and indices. However, this is also an illustration of the conceptual focus and the way gender was approached in specific sub-fields of epidemiology: gender norms measures and discrimination and violence measures conceptualised gender as a catalyst of the individual’s position and experiences in relation to their peers, while gender (in)equality measures focused on the concrete expression of gender imbalances at the society level and, for some of them, on how to address these.

### How do the measures relate to each other?

Our analysis allowed us to see how the measures were developed, on which previous measure or theoretical framework they were based, and how they relate to each other (S4). First, comparing the development of measures over time shows the precedence of research on gender roles and gender-based discrimination. Major developments took place in these fields in the 1960s. Measures on gender (in)equality followed only in the mid-1990s.

With regard to how the measures built on each other, we observed that some seminal research had been the foundation for many measures, for example Nungesser’s work on homosexuality [58] in the gender discrimination and violence category which informed decades of homonegativity research (e.g.[59,60]). The work by Rosenkrantz, Vogel, Bee, Broverman, and Broverman on sex-role stereotypes (1968) [61] has given birth to a series of measures looking at gender role ideology (e.g. [62,63]). Some theories such as the minority stress theory [64] and concepts stemming from anti-racism research [5,65] have laid the conceptual ground for measures in the gender-based discrimination and violence categories [66,67]. Although originating from different areas of research (namely gender ideology and SGM research), the conceptual common ground between the norms and discrimination measures seems to have expanded over decades. The similarity in the dynamics between sexism and heterosexist discrimination finds an origin in the predominance of traditional gender roles and the historical patriarchal advantage given to masculine traits and men “who behave as men” in society. Some instruments in the 1990s and 2000s see the common ground and try to combine the feminist and SGM literatures. For example, Konik and Cortina (2008) explicitly made the link between sexism and heterosexism, hypothesizing that oppressions based on gender and sexual orientation are inherently linked in their measure of workplace harassment [68].

We also extracted a large number of measures which have been shortened, revised, adapted (and in some cases validated) for a specific population or a specific country. On the one hand, this shows the adaptability of the measures to historical, societal and cultural change, and the shared understanding of key concepts in the field. Adaptations to particular cultural contexts take into account historically and culturally relevant dimensions of gender (see Brazil [69] and India [70]), in a move from the very US-centered development of measures to a more worldwide effort to take into account gender related dynamics. The adaptability of gender measures is also visible in the way some measures are modified to specifically fit the needs of given target populations, with for examples measures of internalized homophobia [71] refined further into lesbian-specific measures [72]. On the other hand, the relatively frequent use of non-validated or partially validated tools in different populations/countries may raise problems of consistency, reproducibility and validity, since attitudes toward gender roles, discrimination and (in)equality can differ among these [73].

### Which measure for which research? Reflecting on the analytical and implementation potential of measures—A checklist

Table 3 shows how a selection of measures can be assessed against the checklist’s analytical and practice-based items. To show how the checklist would work on a range of instruments, we selected measures that we believed were representative of their type or category (e.g. Attitudes Toward Women measure [74] for the gender norms category and Social and Institutional Gender Index (SIGI) [55] for the gender (in)equality category), and some which were more distinct from the majority, mainly because they integrated more aspects relevant to the analytical items (e.g. African American Men’s Gendered Racism Stress Inventory (AMGRaSI) [67] in the gender-based discrimination and violence category and Health Care Equality Index [75] in the gender (in)equality category). The checklist can be applied easily to the other measures included in this review when using the detailed information on the measures’ characteristics presented in S4.

**Table 3:** Examples of measures and how to use the checklist.

	Analytical items				Practice-based items		
	Inclusion	Intersectionality	Transformative potential	Multilevel approach	Complexity	Validity	Transferability
**Attitudes Toward Women** *Micro level*, *latent*					✓	✓	✓
**Social and Institutional Gender Index** *Macro level*, *manifest*				✓	✓		✓
**Health Care Equality Index** *Meso level*, *manifest*	✓		✓		✓		
**African American Men’s Gendered Racism Stress Inventory** Micro-level, latent		✓			✓	✓	

Broadly speaking, and similar to the SIGI presented in Table 3, measures describing gender (in)equality mostly relied on standard monitoring data and were harmonised at a global scale enabling a widespread application. They could therefore be considered as practical to use, in terms of length/complexity and transferability. As for their analytical potential, they capture several levels or dimensions, because they are usually made up of subscores or dimensions which refer to different aspects of individuals’ lives and societies they live in (e.g. labour, health, institutions). Similar to most of the macro-level measures, they do not include an intersectional dimension. Finally, in terms of inclusion, the SIGI and similar indices were built on a dichotomy between men and women and do not qualify as gender-inclusive measures. Still aiming at measuring aspects of gender equality, a handful of measures were dedicated to assess healthcare facilities’ policies and practices related to the equity and inclusion of their SGM patients, visitors and/or employees. This is the case of the Health Care Quality Index [75], a US measure which evaluates over 1600 facilities. The Health Care Quality Index has an inclusive approach to gender, acknowledging the needs and rights of SGM, and gender-transformative potential through its direct link with medical practice and how care is delivered.

Measures on gender norms tended to incorporate most practice-based items, especially for researchers interested in primary data collection at the individual level. Many of them were validated (although the validation is limited to specific population groups) and could be considered transferable. For example, the Attitudes Toward Women scale has been validated in different sub-groups of populations (e.g. among adolescents [76] or individuals with migration background [77])), and translated in many languages (e.g. Spanish [78], Turkish [79], Korean [80]). From an analytical point of view, their biggest disadvantage was that the vast majority of gender norms measures reproduced stereotypical assumptions of femininity/masculinity or heteronormativity that did not qualify for inclusive nor gender-transformative research. For example, they tended to include items that situated, sometimes exclusively, women in the private sphere, in their role as housewives, and pictured men as breadwinners evolving in broader social environments.

Measures of gender-based discrimination and violence sometimes incorporated a more complex understanding of discriminatory practices and experiences relating to the analytical items of the checklist. As shown in Table 3, the AMGRaSI scale, applied an intersectional approach, specifically assessing the intersection of gender and race among African-American men. Other measures in this category included different levels of social spaces like friends, families, workplace, sports or community and institutional settings. When considering our practice-based items, it is to be noted that measures like the AMGRaSI scale were validated for a specific populations of interest, so accordingly their transferability to other populations or geographical settings was more limited than non-intersectional measures.

## Discussion

A range of gender measures is readily available for epidemiological research, addressing different levels and dimensions of gender as a social construct. They draw a complex picture of gender as a social determinant of health, highlighting all the ways and the areas in which it influences the lives and health of individuals. Through them, gender can be seen as a “super determinant”, which influences health through norms, discrimination and inequalities.

A striking finding was the limited number of measures which went beyond a traditional, binary understanding of gender. Most of the measures could be qualified as gender specific, or gender sensitive, but only very few were intentionally gender transformative, taking steps to challenge the way gender is described and perceived. Equality, diversity and inclusion are cornerstones of the discourse on gender in the fields of sociology, but also feminist and LGBTQ+ activism. However, the gender measures used in epidemiology tended to not operationalise these concepts and to not fulfil the theory-based criteria that reflected on them in our checklist. For example, some psychometric measures still popular nowadays were carrying on stereotypes and repeating very traditional (or even outmoded) perspectives on gender roles, despite some adaptations to societal changes. Items were limited to picturing men’s role as the breadwinner and women’s role as the main caregiver of the family. Egalitarian gender models with”symmetrical” data on employment and care arrangements were rare. One can see a similar pattern in the operationalisations of gender norms in international and national representative surveys. Here, gender biases are perpetuated by the use of questions that are only asked to one sex/gender, e.g. only women are asked about child care or only the impact of a “working mother” but not a “working father” on children’s well-being is assessed [81,82]. As for the macro-level gender (in)equality measures, they were often just comparing health outcomes for women and men based on sex, in what can be considered an essentialist and reductive approach to gender equality [83]. By conflating gender categories with sex, this type of research carries the risk of jeopardizing the validity of gender analysis in epidemiology [84]. Both types—gender norms measures and gender (in)equality indices—are useful for some aspect of epidemiology, but after 6 decades of reflexion on gender in research, it seems that gender measures do not sufficiently accompany societal changes in real-time, nor anticipate change.

As an example of evolving gender landscape, results from a worldwide Ipso survey conducted in 2019 in nationally representative samples of adults highlight that 40% of respondents disagree with a binary definition of gender, acknowledging the existence of a diversity of gender expressions [85]. These changes also apply to sexual orientation with consequences on gender norms, as seen in another survey, in the UK this time, which shows that in 4 years the number of young people who consider themselves bisexuals has been multiplied by 8 [86].

In this context, one wonders how research can be responsive to rapid societal changes, or even better, proactive in investigating gender norms and their impact on health. Some authors argue that one solution is from the get go to avoid repeating clichés and traditional roles, rather building analysis on existing data/variables related to gender. This allows for example for continuous gender measures on a masculine-feminine spectrum [87,88]. The dichotomy still exists but it is seen as two poles rather than exclusionary options and more importantly, there are not predefined attributes of gender roles. Another advantage is that such measures, based on routinely collected data, can provide faster updates and snapshot of practices and interactions.

A second important finding is that, contrary to what an SDH and intersectional approach would promote, gender measures often did not address the fact that gender is about power. The overwhelming focus on the individual in the gender measures was making it difficult to move beyond a liberal, individualistic view of the world where it is each individual’s own responsibility to attain health and well-being. More measures are needed that consider the structural dimensions of gender [89]. Measures taking inspiration from minority stress theory and critical race theory have more explanatory power from that point of view. The latest literature on gender norms offer also some insight into this. It replaces the individual self in relation to others and aims to articulate norms as constraining expectations set by different levels of interactions (i.e. family/relatives, community, and society) [81]. As we did in our systematic review on gender and migration [90], we renew our call for a more systematic investigation of gender power structures, arguing that meso- and macro-levels gender dimensions deserve being investigated next to individuals’ experiences and perceptions.

To overcome some of these shortcomings on gender in epidemiology, one could draw on closely related fields of study. For example, most of the discrimination measures identified in our review were self-reported, individual-level measures. Yet, it has already been shown in research on racial discrimination and health that “people most affected by discrimination may be least able or willing to say so, even as such experiences may nevertheless affect their health” [20]. Similar patterns have been also recently identified with regard to gendered discrimination [91]. Thus, the exposure to discrimination might be underestimated, and scholars on racial discrimination recommend to also take into account implicit measures, for example by using an adapted version of the Implicit Association Test (IAT) [92]. Yet another alternative suggested by Krieger et al. (2020) promotes the use of experimental exposure to discriminatory practices [89].

Last, it is clear that measures extracted for this review have good potential for implementation. Many are short, or have short versions, have been adapted to different settings or populations, or have been revised over the years. It is also common practice for authors to explicitly take into account the practicality in terms of empirical work of the measures (e.g. number of items) and how they will be used in the future, with different populations and in different contexts. This focus on practicality and implementation is valuable in a field where the multiplication and length of data collection tools can represent a burden for research participants.

### Practical suggestions for future epidemiological research

Despite the limitations inherent to some of the gender measures identified, this review still encourages epidemiologists to make the hypothesized gendered pathways explicit in their research, and to operationalise them with measures of gender, to generate valid scientific research on population health. A majority of the articles published in highly influential epidemiology journals and that refer to “gender” in their title, are actually conflating sex and gender. They conduct absolutely necessary but limiting sex-stratified analyses, missing the potential explanatory power of gender as a social determinant. Future research could make use of the checklist developed in this review to identify suitable measures for a given research question. For example, when investigating differentials in suicide risk, one could choose to implement a gender norm measure (in addition to sex stratification), since it has been shown that traditional notions of masculinity are simultaneously associated with specific expectations and stressors, and attitudes that dismiss symptoms and delay care-seeking [93]. The same steps could be applied to yet another topic, namely school-related injuries among pupils. Sex-stratified analyses do show differential types of injuries for girls and boys; however one could take an extra step and investigate how internalised stereotypes, gender-based discrimination and/or gendered social practices might contribute to explain such differences between not only boys and girls, but also gender non-conforming adolescents [94].

Once the hypothesized gendered pathways are explicit, our checklist assists researchers to reflect critically on the measures available (for example, the Meanings of adolescent masculinity scale (MAMS) [95] for the school injuries example) and those to be applied in own analyses. While it can be easily combined with existing frameworks on how to integrate gender throughout all research phases (e.g. [12,96]), our checklist is fully committed to the actual measurement of gender, including in its most empirical dimension -which has not necessarily been the focus of previous frameworks.

### Strength and limitations

This scoping review approached gender as a social determinant from an intersectional, SDH-informed perspective and included a range of measures, encompassing reflections on sexism, homophobia and gender equality. This goes beyond what has been done so far and allows for a holistic approach towards conceptualising and measuring gender that is useful for epidemiology. However, it also means that some nuance in the types of measures and what they achieve may have been lost for the sake of synthesis in our analysis. Additionally, because of our large scope, we may have missed specific types of measures. This is for example the case in the field of domestic violence, or intimate partner violence: although many measures exist, some of the most common ones did not show up in our initial searches. This may be a reflection of how they are conceptualised, not looking at the gender dimensions of violence, but merely counting events and certain types of manifest expressions of gender-based violence [97]. We included though intimate partner violence measures that had a clear gender dimension (e.g. [98]). The absence of obstetric violence measures, a typical gender issue that has not always been framed as such so far in the epidemiological literature, is also telling [99]. Finally, a strength of our work is that we translated the findings into practical steps for researchers to investigate gender in the future. With a short tool, we highlighted key criteria that can guide reflection and epidemiological research design.

## Conclusions

A range of gender measures are readily available for epidemiological research, addressing different levels and dimensions of gender as a social construct. With our theory-informed, practice-driven scoping review, we highlighted their strengths and limitations and provided analytical tools for researchers interested in conducting quantitative gender research. This user-friendly, empirical approach to measuring gender will hopefully nourish the reflection on gender as a social determinant of health and serve as a basis for more methodological developments in epidemiology.

## Supporting information

S1 File PRISMA-ScR checklist. (DOCX)

S2 File Search strategy. (DOCX)

S3 File Flow diagram. (DOCX)

S4 File Detailed characteristics of the gender measures (extraction table). (XLSX)
