# Supplementary material for: Measurement of gender as a social determinant of health in epidemiology—A scoping review
Source: PLoS One. 2021 Nov 3;16(11):e0259223. doi: 10.1371/journal.pone.0259223 (PMC8565751; doi:10.1371/journal.pone.0259223)
Supplement: S2 File — (DOCX) [file pone.0259223.s002.docx]

**Supplementary material 2: Search queries in electronic databases**

*The initial search was conducted on 19.11.2019 in the three databases. An update was conducted on 11.02.2021 in Pubmed and PsycINFO only as the research team lost institutional access to the Embase database in-between the two searches.*

**Pubmed**

("measure"[Title/Abstract] OR "measurement"[Title/Abstract] OR “instrument”[Title/Abstract] OR “tool”[Title/Abstract] OR "index"[Title/Abstract] OR "scale"[Title/Abstract])

AND

("gender inequality"[Title/Abstract] OR "gender discrimination"[Title/Abstract] OR "gender equity"[Title/Abstract] OR "gender role"[Title/Abstract] OR "gender norm"[Title/Abstract] OR "gender stereotype"[Title/Abstract] OR "gender attitude"[All Fields] OR "gender identity"[Title/Abstract] OR "gender expression"[Title/Abstract] OR "gendered behaviour" [Title/Abstract] OR "masculinity" [Title/Abstract] OR “masculine" [Title/Abstract] OR "femininity" [Title/Abstract] OR “feminine" [Title/Abstract] OR "LGBT" [Title/Abstract] OR "homophobia" [Title/Abstract] OR "transphobia" [Title/Abstract] OR "biphobia" [Title/Abstract] OR "heteronormativity" [Title/Abstract] OR "androgyny" [Title/Abstract] OR "homonegativity" [Title/Abstract] OR "gender gap" [Title/Abstract] OR "Transgender-inclusive" [Title/Abstract] OR “sexism” [Title/Abstract] OR “machismo” [Title/Abstract] OR “misogyny” [Title/Abstract])

**PsycINFO**

("measure" OR "measurement" OR “instrument” OR “tool” OR "index” OR "scale”)

AND

("gender inequality" OR "gender discrimination" OR "gender equity" OR "gender role" OR "gender norm" OR "gender stereotype" OR "gender attitude" OR "gender identity" OR "gender expression" OR "gendered behaviour" OR "masculinity" OR “masculine” OR "femininity" OR “feminine” OR "LGBT" OR "homophobia" OR "transphobia" OR "biphobia" OR "heteronormativity" OR "androgyny" OR "homonegativity" OR "gender gap" OR "Transgender-inclusive" OR “sexism” OR “machismo” OR “misogyny”)

AND

(“health” OR “well-being” OR “mortality” OR "life expectancy" OR “morbidity” OR “risk” OR “disease” OR "physical" OR "psychological" OR "mental health" OR "health inequalities" OR "depression" OR “anxiety” OR “psychosis” OR “stress” OR “minority stress” OR “PTSD” OR “post-traumatic stress syndrome” OR health status OR health equity OR health service OR sexual health OR child’s health OR women’s health OR men’s health OR health behaviour OR health promotion OR health information)

Filter: Population(human), journal type(peer-reviewed journals)

**Embase, MEDLINE**

Query('measure':ab,ti OR 'measurement':ab,ti OR 'instrument':ab,ti OR 'tool':ab,ti OR 'index':ab,ti OR 'scale':ab,ti) AND ('gender inequality':ab,ti OR 'gender discrimination':ab,ti OR 'gender equity':ab,ti OR 'gender role':ab,ti OR 'gender norm':ab,ti OR 'gender stereotype':ab,ti OR 'gender attitude':ab,ti OR 'gender identity':ab,ti OR 'gender expression':ab,ti OR 'gendered behaviour':ab,ti OR 'masculinity':ab,ti OR 'femininity':ab,ti OR 'lgbt':ab,ti OR 'homophobia':ab,ti OR 'transphobia':ab,ti OR 'biphobia':ab,ti OR 'heteronormativity':ab,ti OR 'androgyny':ab,ti OR 'homonegativity':ab,ti OR 'gender gap':ab,ti OR 'transgender-inclusive':ab,ti OR 'sexism':ab,ti OR 'machismo':ab,ti OR 'misogyny':ab,ti) AND [humans]/lim AND [2000-2019]/py
