# Supplementary material for: Measurement of gender as a social determinant of health in epidemiology—A scoping review
Source: PLoS One. 2021 Nov 3;16(11):e0259223. doi: 10.1371/journal.pone.0259223 (PMC8565751; doi:10.1371/journal.pone.0259223)
Supplement: S3 File — (DOCX) [file pone.0259223.s003.docx]

**Supplementary material 3: Flow diagram**

**Eligibility**

**Included**

**Screening**

**Identification**

Records identified through database searching
(n=6777)

Records after duplicates removed
(n=5085)

Note: The number of measures included in the synthesis differ from the number of articles considered relevant for the review. This is due to the fact that many measures have been used in different studies and a single study could include several gender measures.

Additional measures identified through snowballing searches
(n=41)

Measures included in qualitative synthesis
(n=344)

Records excluded
(n=3535)

Full-text articles excluded:

Not measure of gender (n=83)

Full text missing (n=15)

Not quantitative (n=12)

Language (n=4)

Not well described (n=7)

Not health relevant (n=1)

Full-text articles assessed for eligibility
(n=1550)

Records screened
(n=5085)
